# National screening for anxiety and depression in Saudi Arabia 2022

**DOI:** 10.3389/fpubh.2023.1213851

**Published:** 2023-06-27

**Authors:** Abdulhameed Abdullah Alhabeeb, Rashed Abdullah Al-Duraihem, Saeed Alasmary, Zaied Alkhamaali, Nora A. Althumiri, Nasser F. BinDhim

**Affiliations:** ^1^National Centre for Mental Health Promotion, Riyadh, Saudi Arabia; ^2^Saudi Food and Drug Authority, Riyadh, Saudi Arabia; ^3^Informed Decision-Making for Research and Studies, Riyadh, Saudi Arabia; ^4^College of medicine, Alfaisal University, Riyadh, Saudi Arabia

**Keywords:** mental health, depression, anxiety, screening, Saudi Arabia

## Abstract

**Background:**

Mental health disorders, such as major depressive disorder (MDD) and generalized anxiety disorder (GAD), represent a significant public health concern in Saudi Arabia. This study aims to provide a recent mental health screening prevalence, including anxiety and depression screening in the general public and to explore the associated risk factors.

**Methods:**

A cross-sectional study was conducted, employing a phone interview survey with 6,015 participants, using a quota sampling strategy to ensure equal representation of both sexes and administrative regions. The study assessed the prevalence of MDD and GAD risk and examined demographic, socioeconomic, and lifestyle factors associated with these mental health disorders.

**Results:**

The national prevalence of people at risk of MDD and GAD were found to be 12.7 and 12.4%, respectively. Low diagnosis and treatment rates were observed, with only 1.5 and 0.5% of participants currently diagnosed and treated for depression and anxiety, respectively. Risk factors for MDD and GAD included female sex, lower education and income levels, smoking, and waterpipe use. Protective factors included physical activity, participation in volunteering activities and the practice of daily hobbies in the last 30 days.

**Conclusion:**

The relatively high prevalence of MDD and GAD risk and low diagnosis and treatment rates in Saudi Arabia emphasize the need for increased mental health promotion, early detection, and treatment accessibility. The study highlights the importance of addressing modifiable risk factors and fostering protective factors through targeted interventions. Future research should focus on longitudinal associations, potential mediators and moderators, and the development of culturally appropriate and evidence-based interventions to enhance mental health outcomes in the region.

## 1. Introduction

The importance of national screening for mental health cannot be overstated. Mental health issues, particularly depression and anxiety, have become increasingly prevalent in recent years, posing a significant public health concern (1). Early identification and timely intervention have been shown to improve treatment outcomes and reduce the burden of mental health disorders on individuals, families, and society as a whole (2).

National screening programs can help identify individuals at risk for mental health disorders and facilitate their access to appropriate care. In addition, such programs can provide valuable data for researchers and policymakers, allowing them to better understand the prevalence, risk factors, and demographic characteristics of mental health disorders, and to design targeted interventions to address these issues (3). Furthermore, national screening programs can contribute to reducing the stigma associated with mental health disorders, as they underscore the importance of mental health as a public health priority (4).

Despite the increasing recognition of the importance of mental health, many individuals with depression and anxiety remain undiagnosed and untreated. According to the World Health Organization (5), the global prevalence of depression is estimated to be approximately 4.4% and anxiety disorders at about 3.6%. However, it has been estimated that up to 50% of individuals with these conditions do not receive a formal diagnosis or treatment (6).

There are several reasons for this high rate of undiagnosed mental health disorders. First, depression and anxiety symptoms can be non-specific and easily overlooked, particularly in primary care settings (7). Second, stigma and lack of awareness surrounding mental health can prevent individuals from seeking help (8). Finally, limited access to mental health care services, particularly in low-and middle-income countries, can further exacerbate the problem (9).

Saudi Arabia, like many other countries, faces significant challenges in addressing the mental health needs of its population (10, 11). A recent large national screening using the Patient health questionnaire-9 (PHQ-9) and the Generalized Anxiety Disorder 7 (GAD-7) in 2020 showed that the prevalence of Major Depressive Disorders (MDD) is at 13.8% while Generalized Anxiety Disorders were at 11.8% (12).

Several risk factors have been identified for depression and anxiety in Saudi Arabia, including female gender, low income, low educational level, unemployment, and chronic health conditions (12, 13). Furthermore, social factors such as marital status, family structure, and social support can also play a significant role in the development and maintenance of mental health disorders (12, 13).

Given the high prevalence of risk of MDD and GAD in Saudi Arabia and the associated burden on individuals and society, it is crucial to collect recent and frequent national statistics on these mental health disorders. Regularly updated data on the prevalence, risk factors, and demographic characteristics of depression and anxiety can help inform the development and implementation of targeted interventions and policies (1, 12).

Moreover, such data can contribute to raising awareness about mental health issues and combating the stigma associated with these disorders (4). Furthermore, accurate and up-to-date statistics can facilitate international comparisons and enable researchers to better understand the global context of mental health disorders, thereby informing global mental health initiatives and strategies (3).

In conclusion, the importance of national screening for mental health, particularly for depression and anxiety, cannot be overstated. Such programs can help identify individuals at risk, facilitate access to appropriate care, and provide valuable data for researchers, policymakers, and mental health advocates (14). In the context of Saudi Arabia, there is a pressing need for recent and frequent national statistics to inform targeted interventions and policies, reduce stigma, and contribute to the global understanding of mental health issues. Prior research on creating a national mental health screening surveillance system in Saudi Arabia took place in 2020, amid the COVID-19 pandemic. The system underwent testing in four phases, producing consistent and valid results (12, 14, 15). To maintain ongoing mental health monitoring, the National Center for Mental Health Promotion (NCMH) launched an annual program in 2022, utilizing the knowledge and infrastructure established during the 2020 surveillance system. Additionally, the 2020 surveillance system primarily concentrated on epidemic-related factors, such as national lockdowns and social distancing measures. While the new program plans to consider current and future large-scale events, its primary focus lies in addressing demographic and preventive factors, with long-term continuity.

Thus, this study aims to provide a recent mental health screening prevalence, including anxiety and depression screening in the general public and to explore the associated risk factors.

## 2. Materials and methods

### 2.1. Design

This study is a cross-sectional, nationwide mental health screening carried out through computer-aided telephonic interviews, in October–November 2022. The data collectors, who were skilled in conducting telephone interview research, underwent two training sessions to familiarize themselves with the interview manual before conducting the interviews. In this national survey we adopted the same methodology of the Saudi Arabia Mental Health Surveillance System (MHSS) (12, 14) which showed high quality and validity and will allow for comparison with historical data (15). Data collection and management were executed through the ZDataCloud research data collection system for enhanced organization and efficiency (16).

### 2.2. Participants and recruitment

Saudi Arabia residents, adults aged 18 and above were enlisted using a randomly generated phone number list, encompassing all 13 administrative regions of Saudi Arabia. Individuals were reached via telephone up to three attempts. In case of non-response, an alternative potential participant with a comparable demographic background (age, gender, area) was approached. The data collectors sought verbal consent from participants and recorded it in the consent field of the data collection system. If a participant did not give consent, an alternative individual with a similar demographic background (age, gender, area) was approached instead.

### 2.3. Sample size

The SMHNS employed a proportional quota sampling method to attain a balanced distribution of participants, stratified according to age, gender, and region within and among the 13 administrative divisions of Saudi Arabia. Two age categories were formed based on the Saudi median adult age of 36 years (one category consisted of individuals aged 18 to 36 years, while the other comprised those aged 37 years and above). This resulted in a quota of 52 for this national survey, which contributed to enhancing the sample’s diversity and minimizing the likelihood of nonprobability sampling bias.

Based on the desired depth of sub-analysis, we determined the sample size, which involved comparing age and sex groups across regions with a medium effect size of roughly 0.26, 80% power, and a 95% confidence level (17). Consequently, each quota was recommended to have 115 participants, resulting in a total of 460 individuals per region and an overall sum of 5,980 participants as a total sample. Upon fulfilling the quota sample, participants with comparable attributes were not allowed to partake in the research.

### 2.4. Data collection

The quota sampling technique is an automated procedure, as it is managed automatically by the ZDataCloud data collection system without human intervention, which also reduces sampling bias (16). As the data collection system closed the quotas after achieving only the targeted sample and as a group of phone call attempts was happening concurrently, on some occasions, more than one participant could pass the eligibility process at the same time, increasing the sample size above the target for some of the quotas. Thus, a slightly larger sample size maybe recruited for some quotas. Since the research utilized automated electronic data collection and management, no missing values were present; additionally the ZDataCloud incorporates a data integrity verification feature to deter data collectors from inputting incorrect information.

### 2.5. Study variables

The study comprises multiple sections, including demographic data, health lifestyle and behavior, and psychological well-being screening. During the data compilation phase, demographic factors including age, sex, administrative area, educational achievement, income level, and marital status were collected. Furthermore, details regarding other health-related variables, existing depression or anxiety diagnosis, obesity, physical activity, practicing hobbies, volunteering, and smoking habits, were also acquired.

In this study, the primary instrument employed for screening mental health was the Patient Health Questionnaire (PHQ-9) (18–20). The choice of PHQ-9 over other depression assessment methods was based on several factors (1): its validation across different age categories, such as adolescents, adults, and older individuals (12, 21) (2); its consistent performance irrespective of administration mode (e.g., self-reported by patients, administered in person or *via* phone by interviewers, or through touch-screen devices) (21, 22) (3); its proven validity and reliability for depression screening in a Saudi sample (12, 23). Additionally, the PHQ-9 has been employed in numerous global assessments and monitoring systems for mental health evaluation, such as the CDC’s utilization in the United States for the Behavioral Risk Factor Surveillance System and the National Health and Nutrition Examination Survey; this also enables international comparisons (24).

Finally, the Generalized Anxiety Disorder-7 (GAD-7) was employed to screen for anxiety levels, exhibiting strong validity and reliability across multiple studies (15, 25). Furthermore, GAD-7 has proven its validity in screenings of general populations, inclusive of Arabic speakers within the Saudi community (15, 26, 27).

### 2.6. Outcome measures and variables transformation

In order to measure the occurrence of risk of depression within our sample, we applied a score exceeding 10, which demonstrated the most favorable balance between sensitivity, 0.89 (95% CI 0.75 to 0.96), and specificity, 0.89 (95% CI 0.79 to 0.94), based on the combined findings from 10 studies (12, 28).

Regarding the GAD-7, the collective sensitivity and specificity values seemed to be satisfactory at a threshold of 8 [sensitivity: 0.83 (95% CI 0.71–0.91), specificity: 0.84 (95% CI 0.70–0.92)], while cut-off scores ranging from 7 to 10 also exhibited comparable combined sensitivity/specificity estimates (29). Moreover, in relation to the GAD-7 anxiety metric, a score of 10 or higher presented the ideal cut-off point in both the literature and prior research conducted on Saudi demographics (25, 26).

The study also adhered to the World Health Organization’s (WHO) global recommendations for adult physical activity (ages 18–64), which include (1): 75 min of vigorous intensity physical activity (VIPA) per week, or (2) 150 min of moderate intensity physical activity (MIPA) per week (30). Based on the self-reported information gathered from participants through interview questionnaires (e.g., exercise duration, frequency, and intensity per week), two distinct outcome variables were established to determine if the guidelines were met: an adequate level of physical activity (ALPA) (consisting of a minimum of 150 min of MIPA weekly and/or at least 75 min of VIPA weekly) and a suboptimal level of physical activity (LLPA) (falling below 150 min of MIPA and/or 75 min of VIPA).

Other variables were presented as obtained with no transformation processes.

### 2.7. Statistical analysis

The prevalence was reported as proportion of the overall sample. For quantitative variables with a normal distribution, means and standard deviations (SD) are provided, while medians and ranges are used when appropriate. Qualitative variables are displayed as percentages and confidence intervals (CIs) and are compared using Pearson’s chi-square test. Multivariate regression analysis (logistic regression) was used to investigate risk factors related to the susceptibility of Major Depressive Disorder (MDD) or Generalized Anxiety Disorder (GAD).

## 3. Results

### 3.1. Demographics

Of the 9,248 potential participants contacted, 6,015 from the 13 administrative regions of Saudi Arabia completed the phone interview, with a response rate of 65.04%, including those who did not answer on 3 occasions. Out of the total participants as planned in the quota sampling, 50% were female. The mean age was 36.45 (SD 12.45; range: 18–90). Table 1 shows the demographic distribution of the sample.

**Table 1:** Participant demographics in the sample.

Characteristics	*N* (%)
Age Groups	
18–19	164 (2.7)
20–29	1772 (29.5)
30–39	1,662 (27.6)
40–49	1,429 (23.8)
50–59	671 (11.2)
60+	317 (5.3)
Sex	
Male	3,007 (50.0)
Female	3,008 (50.0)
Education Level	
Less than a bachelor’s degree	3,008 (50.0)
Bachelor’s degree and above	3,007 (50)
Income Level	
Less than 5 K SAR/Month	1,610 (26.8)
More than 5 K SAR/Month	3,031 (50.4)
No Stable monthly income	1,374 (22.8)
Marital Status	
Currently married	2,335 (38.8)
Currently not married	3,680 (61.2)
Region	
Asir	461 (7.7)
Baha	460 (7.6)
Eastern region	463 (7.7)
Hail	463 (7.7)
Jazan	461 (7.7)
Al Jouf	461 (7.7)
Madinah	467 (7.8)
Makkah	462 (7.7)
Najran	462 (7.7)
Northern border	467 (7.7)
Qassim	463 (7.7)
Riyadh	462 (7.7)
Tabuk	463 (7.7)

### 3.2. Mental health risks and associated variables

The sample prevalence of people at risk of MDD (PHQ-9—Cut-Off above 10) was 12.7%. The national prevalence of people at risk of GAD (GAD-7—Cut-Off above 10) was 12.4%.

Table 2 shows the association between risk of MDD and all other variables. Overall, the following variables were significantly associated with risk of MDD (sex, region, education, monthly income, smoking cigarette, waterpipe smoking, physical activity, volunteering in the last 30 days, and practicing a daily hobby in the last 30 day).

**Table 2:** Cross-tabulation of all variables with risk of MDD.

Variables	At risk of MDD *n* (%)		Total *n* (%)	*P* value chi-square
	Yes	No		
Sex				0.002
Male	342 (11.4)	2,665 (88.6)	3,007 (50.0)	
Female	424 (14.1)	2,584 (85.9)	3,008 (50.0)	
Age groups (Year)				0.874
18–19	22 (13.4)	142 (86.6)	164 (2.7)	
20–29	229 (12.9)	1,543 (87.1)	1772 (29.5)	
30–39	210 (12.6)	1,452 (87.4)	1,662 (27.6)	
40–49	174 (12.2)	1,255 (87.8)	1,429 (23.8)	
50–59	84 (12.5)	587 (87.5)	671 (11.2)	
60+	47 (14.8)	270 (85.2)	317 (5.3)	
Regions				<0.001
Asir	77 (16.7)	384 (83.3)	461 (7.7)	
Baha	43 (9.3)	417 (90.7)	460 (7.6)	
Eastern region	65 (14.0)	398 (86.0)	463 (7.7)	
Hail	38 (8.2)	425 (91.4)	463 (7.7)	
Jazan	80 (17.4)	381 (82.6)	461 (7.7)	
Al Jouf	51 (11.1)	410 (88.9)	461 (7.7)	
Madinah	63 (13.5)	404 (86.5)	467 (7.8)	
Makkah	71 (15.4)	391 (84.6)	462 (7.7)	
Najran	90 (19.5)	372 (80.5)	462 (7.7)	
Northern border	47 (10.1)	420 (89.9)	467 (7.8)	
Qassim	38 (8.2)	425 (91.8)	463 (7.7)	
Riyadh	63 (13.6)	399 (86.4)	462 (7.7)	
Tabuk	40 (8.6)	423 (91.4)	463 (7.7)	
Education				<0.001
Less than bachelor’s degree	434 (14.4)	2,574 (85.6)	3,008 (50.0)	
bachelor’s degree or above	332 (11.0)	2,675 (89.0)	3,007 (50.0)	
Monthly Income				0.007
Less than 5 K SAR/Month	241 (15.0)	1,369 (85.0)	1,610 (26.8)	
More than 5 K SAR/Month	358 (11.8)	2,673 (88.2)	3,031 (50.4)	
No Stable monthly income	167 (12.2)	1,207 (87.8)	1,374 (22.8)	
Marital Status				
Married	449 (12.2)	3,231 (87.8)	3,680 (61.2)	0.119
Single	317 (13.6)	2018 (86.4)	2,335 (38.8)	
Smoking Cigarette				
Yes	182 (16.2)	942 (83.8)	1,124 (18.7)	<0.001
No	584 (11.9)	4,307 (88.1)	4,891 (81.3)	
Waterpipe Smoking				
Yes	142 (17.6)	667 (82.4)	809 (13.4)	<0.001
No	624 (12.0)	4,582 (88.0)	5,206 (86.6)	
Physical Activity				
ALPA	121 (9.7)	1,128 (90.3)	1,249 (20.8)	<0.001
LLPA	645 (13.5)	4,121 (86.5)	4,766 (79.2)	
Volunteering in the last 30 days				
Yes	75 (9.8)	668 (90.2)	763 (12.7)	0.01
No	691 (13.2)	4,561 (86.8)	5,252 (87.3)	
Practicing a daily hobby in the last 30 day				
Yes	139 (9.9)	1,271 (90.1)	1,410 (23.4)	<0.001
No	627 (13.6)	3,978 (86.4)	4,605 (76.6)	
Grand Total	766	5,249	6,015	

Table 3 shows the association between risk of GAD and all other variables. Overall, the following variables were significantly associated with risk of GAD (sex, region, education, monthly income, smoking cigarette, waterpipe smoking, physical activity, volunteering in the last 30 days, and practicing a daily hobby in the last 30 day).

**Table 3:** Cross-tabulation of all variables with risk of GAD.

Variables	At risk of GAD *n* (%)		Total *n* (%)	*P* value chi-square
	Yes	No		
Sex				0.001
Male	329 (10.9)	2,678 (89.1)	3,007 (500.0)	
Female	418 (13.9)	2,590 (86.1)	3,008 (500.0)	
Age Groups (Year)				0.278
18–19	26 (15.9)	138 (84.1)	164 (20.7)	
20–29	214 (12.1)	1,558 (87.9)	1772 (290.5)	
30–39	194 (11.7)	1,468 (88.3)	1,662 (270.6)	
40–49	176 (12.3)	1,253 (87.7)	1,429 (230.8)	
50–59	87 (13.0)	584 (87.0)	671 (110.2)	
60 +	50 (15.8)	267 (84.2)	317 (50.3)	
Regions				<0.001
Asir	64 (13.9)	397 (86.1)	461 (70.7)	
Baha	41 (8.9)	419 (91.1)	460 (70.6)	
Eastern region	96 (20.7)	367 (79.3)	463 (70.7)	
Hail	43 (9.3)	420 (90.7)	463 (70.7)	
Jazan	78 (16.9)	383 (83.1)	461 (70.7)	
Al Jouf	53 (11.5)	408 (88.5)	461 (70.7)	
Madinah	51 (10.9)	416 (89.1)	467 (70.8)	
Makkah	60 (13.0)	402 (87.0)	462 (70.7)	
Najran	108 (23.4)	354 (76.6)	462 (70.7)	
Northern border	37 (7.9)	430 (92.1)	467 (70.8)	
Qassim	24 (5.2)	439 (94.8)	463 (70.7)	
Riyadh	48 (10.4)	414 (89.6)	462 (70.7)	
Tabuk	44 (9.5)	419 (90.5)	463 (70.7)	
Education				0.039
Less than bachelor’s degree	400 (13.3)	26.8 (86.7)	3,008 (500.0)	
bachelor’s degree or above	347 (11.5)	2,660 (88.5)	3,007 (500.0)	
Monthly Income				0.041
Less than 5 K SAR/Month	224 (13.9)	1,386 (86.1)	1,610 (260.8)	
More than 5 K SAR/Month	346 (11.4)	2,685 (88.6)	3,031 (500.4)	
No Stable monthly income	117 (12.9)	1,197 (87.1)	1,374 (220.8)	
Marital Status				
Married	466 (12.7)	3,214 (87.3)	3,680 (610.2)	0.471
Single	281 (12.0)	2054 (88.0)	2,335 (380.8)	
Smoking Cigarette				
Yes	167 (14.9)	957 (85.1)	1,124 (180.7)	0.006
No	580 (11.9)	4,311 (88.1)	4,891 (810.3)	
Waterpipe Smoking				
Yes	133 (16.4)	676 (83.6)	809 (130.4)	<0.001
No	614 (11.8)	4,592 (88.2)	614 (860.6)	
Physical Activity				
ALPA	130 (10.4)	1,119 (89.6)	1,249 (200.8)	0.015
LLPA	617 (12.9)	4,149 (87.1)	4,766 (790.2)	
Volunteering in the last 30 days				
Yes	64 (8.4)	699 (91.6)	763 (120.7)	<0.001
No	683 (13.0)	4,569 (87.0)	5,252 (870.3)	
Practicing a daily hobby in the last 30 days				
Yes	137 (9.7)	1,273 (90.3)	1,410 (230.4)	<0.001
No	610 (13.2)	3,995 (86.8)	4,605 (760.6)	
Grand Total	766	5,249	6,015	

Only 93 (1.5%) participants are currently diagnosed with depression and on-treatment, and only 32 (0.5%) participants are currently diagnosed with anxiety and on-treatment.

Finally, logistic regression model was preformed including all the variables to further explore which of the variables are more likely to be associated with risk of MDD and GAD. As shown in Table 4, Sex, Region, Monthly Income, Education, practicing a daily hobby in the last 30 days, volunteering in the last 30 days, smoking cigarette, waterpipe smoking, and physical activity were significantly associated with MDD risk.

**Table 4:** MDD risk regression model.

	Value of *p*	Exp(B)	95% C.I. for EXP(B)	
			Lower	Upper
Sex (Female)	**<0.001**	1.472	1.243	1.745
Region (Qassim as reference)	**<0.001**			
Al Jouf	0.358	1.233	0.789	1.927
Northern border	0.612	1.124	0.715	1.768
Tabuk	1.000	1.000	0.626	1.596
Hail	0.681	0.906	0.564	1.454
Madinah	**0.022**	1.650	1.074	2.534
Makkah	**0.003**	1.914	1.254	2.920
Riyadh	**0.022**	1.651	1.074	2.536
Eastern region	**0.010**	1.747	1.140	2.678
Baha	0.741	1.081	0.682	1.712
Asir	**<0.001**	2.099	1.384	3.183
Jazan	**0.001**	2.011	1.326	3.049
Najran	**<0.001**	2.601	1.730	3.911
Monthly Income (less than 5 K as reference)	**0.018**			
More than 5 K SAR/Month	**0.033**	0.808	0.663	0.983
No Stable monthly income	**0.008**	0.745	0.599	0.928
Education (bachelor’s degree or above)	**0.002**	0.771	0.654	0.908
Practicing a daily hobby in the last 30 days (Yes)	**0.006**	0.749	0.610	0.920
Volunteering in the last 30 days (Yes)	**0.008**	0.703	0.542	0.914
Smoking Cigarette (Yes)	**<0.001**	1.485	1.201	1.835
Waterpipe Smoking (Yes)	**0.002**	1.429	1.141	1.790
Physical Activity (ALPA)	**0.038**	0.798	0.645	0.987
Marital Status (Married)	0.245	0.895	0.743	1.079
Age	0.348	1.003	0.996	1.010

Bold indicates significant associations.

On the other hand, as shown in Table 5, sex, region, monthly income, practicing a daily hobby in the last 30 days, volunteering in the last 30 days, smoking cigarette, and waterpipe smoking, were significantly associated with MDD risk.

**Table 5:** GAD risk regression model.

	Value of *p*	Exp(B)	95% C.I. for EXP(B)	
			Lower	Upper
Sex (Female)	**<0.001**	1.439	1.211	1.711
Region (Qassim as reference)	**<0.001**			
Al Jouf	**0.002**	2.207	1.332	3.656
Northern border	0.195	1.423	0.834	2.428
Tabuk	**0.018**	1.868	1.113	3.136
Hail	**0.035**	1.749	1.040	2.942
Madinah	**0.002**	2.212	1.333	3.672
Makkah	**0.000**	2.593	1.578	4.260
Riyadh	**0.006**	2.059	1.235	3.434
Eastern region	**0.000**	4.734	2.954	7.588
Baha	**0.049**	1.693	1.003	2.859
Asir	**0.000**	2.854	1.745	4.666
Jazan	**0.000**	3.405	2.102	5.516
Najran	**0.000**	5.524	3.463	8.813
Monthly Income (less than 5 K as reference)	**0.022**			
More than 5 K SAR/Month	**0.006**	0.751	0.613	0.920
No Stable monthly income	0.201	0.866	0.695	1.080
Education (bachelor’s degree or above)	0.287	0.914	0.774	1.079
Practicing a daily hobby in the last 30 days (Yes)	**0.009**	0.758	0.616	0.933
Volunteering in the last 30 days (Yes)	**0.001**	0.619	0.469	0.818
Smoking Cigarette (Yes)	**0.001**	1.440	1.157	1.791
Waterpipe Smoking (Yes)	**0.004**	1.405	1.115	1.772
Physical Activity (ALPA)	0.417	0.917	0.743	1.131
Marital Status (Married)	0.369	1.092	0.902	1.322
Age	0.226	1.004	0.997	1.012

Bold indicates significant associations.

## 4. Discussion

In this cross-sectional study, we achieved a response rate of 65.04% among the 6,015 participants, which is considered satisfactory for a phone interview survey (31). The quota sampling strategy employed in this study ensured an equal representation of both sexes, with 50% of the participants being female. The mean age of participants was 36.45, similar to the general population in Saudi Arabia (32).

The sample prevalence of people at risk of MDD and GAD were found to be 12.7 and 12.4% respectively, which is consistent with previous studies conducted in Saudi Arabia and overall lower than many other countries (12). Although this study results were similar to the results of the 2020 MHSS which were conducted during the COVID-19 epidemic, with the lack of more historical data we could not determine why the results are similar, thus, with the continuity of this national screening program over the coming years more understanding of mental health indicators could be generated. It is important to note that only a small proportion of participants were currently diagnosed and on treatment for depression (1.5%) and anxiety (0.5%), which highlights the potential underdiagnosis and undertreatment of mental health disorders in this population. This assumption is supported by the global prevalence of depression in 5% of adults, with 4% in men and 6% in women (33). Furthermore, a national screening study conducted in 2010 revealed that the 12-month prevalence of any DSM-IV/CIDI disorder is 20.2%, with anxiety disorders are the most common type of disorder, with a lifetime prevalence of 12.3%, followed by mood disorders at 6.8% (34).

Our findings show that sex, region, education, monthly income, smoking cigarette, waterpipe smoking, physical activity, volunteering in the last 30 days, and practicing a daily hobby in the last 30 days were significantly associated with the risk of MDD and GAD. These results are in line with previous research that identified similar factors as predictors of mental health disorders (12, 35, 36).

In our study, females showed a higher risk for both MDD and GAD, which is consistent with previous literature (12, 37). Due to the limited number of prior studies examining regional differences in mental health prevalence, pinpointing a logical explanation proves challenging. However, potential reasons for these disparities may include variations in access to mental health services, cultural factors, socioeconomic differences, or it could be that regional classification is simply a random grouping variable with no real association to mental health (38). Moreover, there is limited information in the literature available on the association between region or city and mental health indicators such as depression or anxiety prevalence. However, existing studies do suggest that regional differences may exist in mental health prevalence (38). The social gradient, which is the relationship between social and economic factors and health outcomes, impacts both the risk of disorder and access to services, ultimately influencing mental health outcomes (38).

The association between lower education and income levels with increased risk of MDD and GAD has been well-documented (39, 40).

Smoking, waterpipe use, and physical inactivity were also significantly associated with increased risk of MDD and GAD, which is consistent with the literature (12, 41, 42). Interestingly, our study discovered a potential link between engaging in volunteer work or pursuing a daily hobby within the past 30 days and a reduced likelihood of being at risk for Major Depressive Disorder (MDD) and Generalized Anxiety Disorder (GAD). Although this cross-sectional study cannot establish causality, the results may suggest that involvement in such activities could provide behavioral activation and protective effects on mental health. Alternatively, individuals with MDD or GAD might be less able to participate in volunteer work, daily hobbies, or physical activities due to the impact of their mental condition (43–46).

Our findings contribute to the growing body of evidence on mental health risks in Saudi Arabia and the Middle East. The high prevalence of MDD and GAD risk, coupled with low diagnosis and treatment rates, calls for increased efforts in mental health promotion, early detection, and treatment accessibility. Additionally, our study highlights the importance of addressing modifiable risk factors such as smoking, physical inactivity, and socioeconomic disparities through targeted interventions. Nonetheless, it is worth noting that in 2019, Saudi Arabia established the NCMH to spearhead mental health promotion and prevention initiatives. In 2021, over 3,500 pages of mental health awareness content were published by NCMH, targeting prevention programs in schools, universities, and workplaces. Collaboration with educational institutions led to conferences, lectures, and teacher training sessions (47, 48). University programs included mental health promotion groups, student support centers, and over 650 awareness activities (47, 48). Workplace initiatives involved mental health first aid training, awareness sessions, and mental health ambassadors (47, 48). Four digital mental health platforms, including NCMH’s Qareboon app, provided thousands of free consultations (47, 48). NCMH also conducted workshops for mental health professionals, focusing on policies, regulations, disaster response, and specialized skills (47, 48).

Future research should explore longitudinal associations between these factors and mental health outcomes, as well as potential mediators and moderators of these relationships. In addition, there is a need for more comprehensive assessments of mental health disorders and their determinants in the region, which will inform the development and implementation of culturally appropriate and evidence-based interventions.

Limitations of the study include the cross-sectional design, which prevents the establishment of causal relationships between variables. Additionally, the use of self-report measures may introduce response bias. Although our study utilized quota sampling, which can be associated with selection bias risks, it was chosen over random probability sampling due to its previously proven consistency and sensitivity to mental health screening in Saudi Arabia and lower costs (15). Furthermore, employing a proportionally large sample and 52 quotas helps mitigate selection bias (31, 49, 50). Currently, in Saudi Arabia, generating a random national-level sample is only feasible through household surveys, which also have limitations caused by cultural factors. Additionally, the utilization of the ZDataCloud research data collection and governance system improved the quota sampling process by eliminating human selection bias. This enhancement as shown in the results led to a well-balanced sample across the 52 quotas, ensuring a more accurate representation of the targeted population. Nonetheless, the study provides valuable insights into the mental health risks and associated variables in the Saudi Arabian population, contributing to the development of effective mental health interventions and policies.

## 5. Conclusion

In conclusion, this cross-sectional study reveals a significant prevalence of individuals at risk for MDD and GAD in Saudi Arabia, and potential underdiagnosis and undertreatment of these mental health disorders. Our findings confirm the association of various factors such as sex, region, education, income, smoking, physical activity, and engagement in hobbies or volunteering with the risk of MDD and GAD. These results underline the need for targeted interventions addressing modifiable risk factors and promoting mental health awareness and treatment accessibility in Saudi Arabia. Future research should investigate longitudinal associations and comprehensive assessments of mental health disorders to inform culturally appropriate, evidence-based interventions. Despite the study’s limitations, it offers valuable insights for developing effective mental health policies and strategies in Saudi Arabia and the Middle East.

## Data availability statement

The raw data supporting the conclusions of this article will be made available by the authors, without undue reservation.

## Ethics statement

The studies involving human participants were reviewed and approved by Sharik Association for Health Research’s ethics committee (Approval no. 04–2022). The patients/participants provided their written informed consent to participate in this study.

## Author contributions

NA and NB participated in the conceptual design and formulation of the research questions. All authors participated in the development and review of the manuscript.

## Funding

This study was funded by the National Center for Mental Health Promotion.

## Conflict of interest

The authors declare that the research was conducted in the absence of any commercial or financial relationships that could be construed as a potential conflict of interest.

## Publisher’s note

All claims expressed in this article are solely those of the authors and do not necessarily represent those of their affiliated organizations, or those of the publisher, the editors and the reviewers. Any product that may be evaluated in this article, or claim that may be made by its manufacturer, is not guaranteed or endorsed by the publisher.
